# Facial Nerve Palsy in Malignant Otitis Externa Hospitalizations: Outcomes and Risk Factors

**DOI:** 10.1002/ohn.70117

**Published:** 2026-01-08

**Authors:** Emma R. Thompson, Anthony M. Saad, Sean Z. Haimowitz, Nour H. Abdel‐Azim, Kevin Wong, Andrey Filimonov

**Affiliations:** ^1^ Rutgers New Jersey Medical School Department of Otolaryngology–Head and Neck Surgery Newark New Jersey USA; ^2^ Department of Otolaryngology Loma Linda University Health Loma Linda California USA; ^3^ Department of Otorhinolaryngology–Head and Neck Surgery University of Pennsylvania Philadelphia Pennsylvania USA

**Keywords:** facial nerve palsy, malignant otitis externa, skull‐based osteomyelitis

## Abstract

**Objective:**

To provide a description of the occurrence of facial nerve palsy in hospitalizations for malignant otitis externa and to compare the demographic characteristics, hospital traits, comorbidities, and outcomes between patients with and without palsy.

**Study Design:**

Retrospective cross‐sectional database study.

**Setting:**

2016 to 2022 National Inpatient Sample (NIS).

**Methods:**

Hospitalizations for malignant otitis externa were identified using ICD‐10 diagnosis codes, and facial nerve palsy was identified using secondary diagnosis codes. Weighted discharge level rates of facial nerve palsy were calculated, and demographics, comorbidities, and outcomes were compared between patients with and without facial nerve palsy.

**Results:**

695 (12.4%) of 5,585 MOE inpatient stays involved facial nerve palsy. Stays involving palsy were significantly more likely to occur in older patients (71.16 ± 13.4 years vs 56.07 ± 21.1 years, *P* < .001), in men (70.7% vs 55.6%, *P* < .001), have a higher number of diagnoses (17.51 vs 13.2, *P* < .001) and chronic conditions (7.91 vs 7.01, *P* = .001) than stays without palsy. Diabetes with chronic complications, complicated and uncomplicated hypertension, and peripheral vascular disease were all significantly more common within the palsy group. Hospitalizations with a palsy diagnosis were associated with longer hospital stays, (7.80 ± 7.5 days vs 5.21 ± 6.8 days, *P* < .001), with smaller proportions of stays resulting in routine discharge.

**Conclusion:**

One in eight US inpatient stays for MOE involved facial nerve palsy, which was associated with older age, advanced comorbidities, longer hospitalizations, and increased care needs following discharge. These findings support the association of facial nerve palsy with heightened comorbidity burden.

Malignant otitis externa (MOE) is an aggressive infection of the external auditory canal that can extend to the temporal bone and skull base, leading to substantial morbidity and mortality.^1^ It is most often caused by *Pseudomonas aeruginosa* and occurs predominantly in older adults with diabetes.^2^

Complications arise when the infection traverses the osteocartilaginous junction and fissures of Santorini, reaching the skull base and adjacent cranial nerves.^3^ Among the resulting cranial neuropathies, facial nerve palsy is the most frequent and can have severe functional and psychosocial consequences for affected patients.^4^

The facial nerve's course through the stylomastoid foramen places it in close proximity to known routes of infectious spread, leaving it particularly vulnerable to damage.^4^ Proposed mechanisms include direct neurotoxicity from bacterial toxins and contiguous spread with subsequent bony erosion, resulting in direct nerve involvement.^4^ Additionally, iatrogenic injury to the nerve can occur during mastoidectomies, which are sometimes performed for advanced MOE.^5^, ^6^, ^7^

Patients with facial nerve palsy experience facial asymmetry, ocular complications, impaired speech, and oral competence, all of which can cause considerable psychosocial distress.^8^, ^9^, ^10^ These functional impairments, coupled with the need to take additional time off for treatment of facial nerve palsy, can result in decreased work productivity and contribute to long‐term disability, incurring additional healthcare costs and financial burdens for affected patients.^11^, ^12^, ^13^ Identifying risk factors for facial nerve palsy in hospitalizations for MOE could help identify high‐risk individuals, facilitating earlier interventions, and potentially improving both functional and economic outcomes.

For clinicians, the risk of facial nerve palsy in hospitalizations for MOE is not well established. Reported incidence rates vary, with a retrospective review from Tehran finding a rate of 26%; a case control study from the same region reporting a rate of 32.4%; and a retrospective review from Tunisia reporting 14.9%.^14^, ^15^, ^16^ Overall, there is a paucity of large, multi‐institutional studies providing rates within the United States population, limiting clinicians’ ability to provide accurate counseling on the expected level of risk.

Facial nerve palsy has also garnered international interest as a potential marker of poor prognosis for MOE, thought to reflect increased disease severity. However, existing evidence for this also comes almost entirely from small, non‐US cohorts and has yielded mixed results. An international chart review found that patients with MOE who presented with facial nerve palsy had higher values of inflammatory markers, more frequent rates of bone osteolysis, greater rates of isolation of fungal pathogens, higher complication rates, and lower rates of disease resolution compared to those without palsy.^16^

Similarly, a retrospective study out of Israel found that facial nerve palsy was an independent predictor of disease‐specific mortality, with an associated threefold increased risk of death within the first year of MOE.^17^ In contrast, a retrospective study from Iran involving over 100 patients found no significant differences in the comorbidities, clinical presentations, laboratory values, or treatment courses between those with and without facial nerve palsy.^14^ Another small Tehran series found no significant differences in hospital course or laboratory findings but did note that recovery from palsy was positively correlated with survival.^18^ A Serbian retrospective review found that patients with facial nerve palsy were more likely to have longer hospital stays and to be readmitted compared to other patients with MOE.^19^ Taken together, these conflicting findings, combined with small sample sizes at single sites, highlight the need for larger, nationally representative studies to clarify the prognostic significance of facial nerve palsy in patients with MOE in the United States.

Accordingly, this study will characterize the proportion of hospitalizations for MOE associated with facial nerve palsy, and identify factors linked to these admissions, recognizing that the National Inpatient Sample captures hospitalizations rather than unique patients. It will also examine the comorbidities and clinical outcomes associated with facial nerve palsy. By utilizing a database that contains a large, national cohort of US patients, our findings aim to support future risk stratification, guide patient counseling, and optimize management strategies.

## Methods

A Non‐Human Research Exemption was received by the Institutional Review Board in New Brunswick, NJ (Pro2025001572). Our study utilized the 2016 to 2022 National Inpatient Sample (NIS).^20^ The NIS is a part of the Healthcare Cost and Utilization Project (HCUP), and is a large, nationally representative public‐use database that contains information on patient demographics, hospital characteristics, and a variety of outcomes for each inpatient stay. Because the NIS captures hospitalizations and may include repeat patients if they were hospitalized more than once for MOE, all analyses were conducted at the hospitalization (stay) level.

Stays for MOE were identified using the primary diagnosis codes for each hospitalization. These were defined as stays with primary diagnoses of H60.20 (malignant otitis externa), H60.21 (malignant otitis externa, right ear), H60.22 (malignant otitis externa, left ear), and H60.23 (malignant otitis externa, bilateral), all of which are International Classification of Diseases, Tenth Revision (ICD‐10) codes describing MOE.^21^ Stays with other primary diagnoses were excluded.

Among MOE stays, cases of facial nerve palsy were identified using the secondary diagnosis fields. Stays with one or more of the following secondary diagnoses were categorized as the “facial nerve palsy” group: ICD‐10 codes G51.0 (Bell's palsy), G51.8 (other specified disorders of facial nerve), and G51.9 (unspecified disorder of facial nerve).^21^ The other codes within this group represent disease processes that are not specific to our study's interest, and were not included (G51.1, G51.2, G51.3, and G51.4).^21^ Stays for MOE without any of the three secondary diagnosis codes were categorized into the “no facial nerve palsy” group.

Elixhauser comorbidities were derived using HCUP's CMR v2025.1 software in SAS (version 9.4).^22^ Because diagnosis‐level present on admission (POA) indicators were not available across all years of the dataset, comorbidity categories that rely heavily on POA status or complex code combinations were excluded. The Elixhauser summary indices (in‐hospital mortality and 30‐day readmission) were also not evaluated because they require all 38 comorbidity measures, necessitating valid POA information.

Analyses were conducted using SPSS (version 29.0.2.0). Descriptive statistics were generated for each variable. All analyses were weighted using the NIS discharge weight (DISCWT) to generate nationally representative, stay‐level estimates. Categorical variables were summarized using frequencies and compared across groups using a Pearson's chi‐squared test or a Fisher's exact test as appropriate, Z tests were used to compare the proportions of discharge outcomes between groups.

Given the large sample size and similar variances, weighted independent‐sample *t*‐tests were used to compare the mean number of chronic conditions and number of diagnoses on record between groups, reporting 95% confidence intervals. After descriptive statistics were obtained, length of stay (LOS) was log‐transformed using log10(LOS + 1) prior to conducting linear regression; exponentiated coefficients were used to represent percentage differences between groups. To evaluate whether facial nerve palsy was independently associated with log‐transformed LOS, a multivariable linear regression model adjusting for age, sex, and several significant comorbidities (diabetes with chronic complications, complicated and uncomplicated hypertension, obesity, depression, and peripheral vascular disease) as well as hospital characteristics was performed. Model assumptions were assessed through inspection of residuals, and variance inflation factors were used to evaluate multicollinearity. Statistical significance was set at *P* < .05 for all tests performed.

## Results

A total of 1256 unweighted, 5585 weighted inpatient stays for MOE were identified, of which 139 unweighted (11.07%) and 695 weighted stays (12.44%) had facial nerve palsy. Stays with facial nerve palsy diagnoses were more likely to involve male patients (70.7% vs 55.6% among discharges without facial palsy, *P < .001) and were associated with older patient ages (71.16 ± 13.4 years vs 56.07 ± 21.1 years, *P* < .001, Table 1). While a majority of patients in both groups were White, racial distributions also differed significantly between groups, with more Black patients in the no palsy group (16.4% vs 12.3%) and more Hispanic patients in the palsy group (24.6% vs 20.9%, *P *= .007, Table 1).

**Table 1:** Patient Demographics

	Facial nerve palsy	No palsy	*P*‐value
Mean age	71.16 (SD 13.4)	56.07 (SD 21.1)	<.001*
Sex			<.001*
Female	29.3% (205)	44.4% (2480)	
Male	70.7% (495)	55.6% (3100)	
Race			.007*
White	55.8% (385)	54.0% (2950)	
Black	12.3% (85)	16.4% (895)	
Hispanic	24.6% (170)	20.9% (1140)	
Asian or Pacific Islander	2.9% (20)	3.3% (180)	
Native American	0% (0)	0.6% (35)	
Other	4.3% (30)	4.8% (265)	

* Statistically significant.

There was regional variation in the distribution of hospitalizations, with relatively higher proportions of facial nerve palsy stays occurring in the Midwest (26.4% vs 19.9%) and West (22.9% vs 15.4%), and lower proportions in the South (32.1% vs 42.2%, *P *< .001, Table 2). Hospitalizations with facial palsy were also more frequently treated in large hospitals (65.0% vs 53.9%, *P* < .001), while other hospital characteristics—including census division, ownership, and urban‐rural denominations—were broadly similar between groups despite statistical significance (Table 2).

**Table 2:** Hospital Characteristics of MOE Inpatient Stays

	Facial nerve palsy % (n)	No palsy % (n)	*P*‐value
Region of hospital			<.001*
Northeast	18.6% (130)	22.5% (1255)	
Midwest	26.4% (185)	19.9% (1110)	
South	32.1% (225)	42.2% (2355)	
West	22.9% (160)	15.4% (860)	
Location and teaching status			.136
Rural	7.1% (50)	5.6% (310)	
Urban nonteaching	13.6% (95)	15.3% (855)	
Urban teaching	79.3% (555)	79.1% (4415)	
Census Division of hospital (STRATA)			<.001*
New England	4.3% (30)	4.5% (250)	
Middle Atlantic	14.3% (100)	18.0% (1005)	
East North Central	17.1% (120)	15.1% (840)	
West North Central	9.3% (65)	4.8% (270)	
South Atlantic	12.9% (90)	25.1% (1400)	
East South Central	9.3% (65)	5.1% (285)	
West South Central	10.0% (70)	12.0% (670)	
Mountain	3.6% (25)	3.9% (215)	
Pacific	19.3% (135)	11.6% (645)	
Relative bed size category of hospital (STRATA)			<.001*
Small	14.3% (100)	19.2% (1070)	
Medium	20.7% (145)	27.0% (1505)	
Large	65.0% (455)	53.9% (3005)	
Control/ownership of hospital (STRATA)			<.001*
Government, nonfederal	17.9% (125)	14.3% (800)	
Private, non‐profit	75.0% (525)	74.3% (4145)	
Private, invest‐own	7.1% (50)	11.4% (635)	
Patient Location: NCHS Urban‐Rural Code			<.001*
“Central” counties of metro areas of >=1 million population	33.6% (235)	35.3% (1960)	
“Fringe” counties of metro areas of >=1 million population	18.6% (130)	25.1% (1395)	
Counties in metro areas of 250,000‐999,999 population	19.3% (135)	20.4% (1130)	
Counties in metro areas of 50,000‐249,999 population	7.9% (55)	7.2% (400)	
Micropolitan counties	13.6% (95)	7.7% (430)	
Not metropolitan or micropolitan counties	7.1% (50)	4.2% (235)	

* Statistically significant, *P* < .05.

There were several statistically significant differences in medical comorbidities between groups (Table 3). Stays with facial nerve palsy were significantly more likely to have diabetes with chronic complications (63% vs 49%, *P* < .001), complicated hypertension (33.3% vs 25.8%, *P* = .001), uncomplicated hypertension (50.6% vs 38.0%, *P* = .001), and peripheral vascular disease (8.6% vs 2.4%, *P* < .001). Palsy was not associated with a different likelihood of diabetes without chronic complications, defined as ophthalmic, renal, neurological, or peripheral circulatory complications (11.1% vs 12.1%, *P* = .554).^22^ Other comorbidities were significantly less common among hospitalizations with facial nerve palsy, including depression (8.6% vs 13.2%, *P* = .009), lymphoma (0% vs 1.1%, *P* = .028), and obesity (13.6% vs 23.6%, *P* < .001).

**Table 3:** Medical Comorbidities

	Facial nerve palsy % (n)	No palsy % (n)	*P*‐value
AIDS	0.0% (0)	0.8% (25)	.105
Cancer—leukemia	1.2% (5)	1.4% (45)	.768
Cancer—lymphoma	0.0% (0)	1.1% (35)	.028*
Cancer—metastatic	1.2% (5)	0.8% (25)	.377
Cancer—solid without metastasis	2.5% (10)	2.0% (65)	.577
Chronic pulmonary disease	17.3% (70)	15.7% (500)	.426
Diabetes with chronic complications	63% (255)	49% (1555)	<.001*
Diabetes without chronic complications	11.1% (45)	12.1% (385)	.554
Dementia	4.9% (20)	4.3% (135)	.523
Depression	8.6% (35)	13.2% (420)	.009*
Hypertension, complicated	33.3% (135)	25.8% (820)	.001*
Hypertension, uncomplicated	50.6% (205)	38.0% (1205)	.001*
Obesity	13.6% (55)	23.6% (750)	<.001*
Peripheral Vascular Disease	8.6% (35)	2.4% (75)	<.001*
Number of diagnoses on this record	17.51 (SD 6.8)	13.2 (SD 6.8)	<.001*, CI = −3.8 to −4.9
Number of chronic conditions	7.91 (SD 3.83)	7.01 (SD 4.01)	<.001*, CI = 0.35‐1.46

* Statistically significant, *P* < .05.

Discharges associated with facial palsy had a higher mean number of ICD‐10 diagnosis codes associated with their stay (17.51 ± 6.8) compared to discharges without facial palsy (13.2 ± 6.8, *P* < .001), with a weighted mean difference of 4.35 diagnoses (95% CI: −3.9 to 4.86, Table 3). They also had a higher mean number of chronic conditions (7.91 ± 3.83) compared with those without facial palsy (7.01 ± 4.01, *P* < .001), with a weighted mean difference of 0.90 chronic conditions (95% CI: 0.35‐1.46).

Length of stay also differed significantly between groups. After log10 transformation, facial nerve palsy was associated with a 49% longer LOS (*B* = 0.396, 95% CI 0.283‐0.508, *P* < .001). The means for unadjusted LOS were 7.80 ± 7.5 days in facial palsy discharges versus 5.21 ± 6.8 days in non‐facial palsy discharges. A multivariable linear regression using log‐transformed LOS adjusting for age, sex, race, significant comorbidities, and hospital characteristics showed that hospitalizations with facial nerve palsy were associated with a 28% longer length of stay compared to hospitalizations without facial nerve palsy (*B* = 0.243, 95% CI: 0.179‐0.308, *P *< .001, Table 4). Older age, diabetes with chronic complications, complicated and uncomplicated hypertension, lymphoma, larger hospital size, and race were also significant predictors of increased LOS, while obesity was associated with a shorter stay. Sex, peripheral vascular disease, and hospital geographic region were not significant predictors. Variance inflation factors were examined to assess multicollinearity; no concerning collinearity was observed among predictors‐the variables of interest remained stable with VIF values near 1.

**Table 4:** Multivariate Regression of Log Transformed Length of Stay

Variable	*B*	Std error	Beta	*t*	*P*‐value	95% CI
Intercept	1.184	0.058	‐	20.328	<.001*	1.069‐1.298
Facial nerve palsy	0.243	0.033	0.118	7.37	<.001*	0.179‐0.308
Hypertension, complicated	0.206	0.033	0.14	6.277	<.001*	0.142‐0.270
Hypertension, uncomplicated	0.057	0.027	0.042	2.11	.035*	0.004‐0.109
Diabetes w/chronic complications	0.171	0.024	0.131	7.223	<.001*	0.125‐0.218
Lymphoma	0.22	0.103	0.033	2.13	.033*	0.018‐0.423
Obesity	−0.091	0.025	−0.058	−3.614	<.001*	−0.140 to −0.042
Peripheral vascular disease	0.036	0.059	0.009	0.6	.548	−0.081 to 0.152
Census Division of hospital	−0.009	0.014	−0.033	−0.626	.531	−0.036 to 0.018
Region of hospital	−0.052	0.033	−0.082	−1.583	.114	−0.117 to 0.012
Relative bed size category	0.097	0.013	0.114	7.262	<.001*	0.071‐0.123
Age (years)	0.004	0.001	0.117	6.096	<.001*	0.002‐0.005
Sex (female)	−0.04	0.022	−0.03	−1.85	.064	−0.083 to 0.002
Race (uniform)	0.019	0.008	0.038	2.369	.018*	0.003‐0.035

Abbreviation: CI, confidence interval.

* Statistically significant, *P* < .05.

Discharge disposition also differed significantly between groups (*P *< .001, Table 5). Hospitalizations with an associated diagnosis of facial nerve palsy were less likely to have a routine discharge compared to MOE discharges without palsy (46.4% vs 67.8%). A greater share of palsy‐associated discharges were transferred to another type of facility (21.4% vs 9.7%) or required home health care services (28.6% vs 17.5%). Very few discharges in either group involved short‐term hospital transfer (3.6% vs 2.2%), leaving against medical advice (0% vs 2.5%), or death (0% vs 0.4%).

**Table 5:** Outcomes

	Facial nerve palsy	No palsy	*P*‐value
Death during hospitalization	0% (0)	0.4% (20)	.157
Elective Admission	8.6% (60)	7.3% (405)	.221
	Facial nerve palsy	No facial nerve palsy	*P*‐value
Disposition of Patient			<.001*
Routine	46.4% (325)_a_	67.8% (3785)_a_	
Short‐term hospital	3.6% (25)_b,c_	2.2% (120)_b,c_	
Skilled Nursing Facility (SNF)	0% (0)	0% (0)	
Intermediate Care Facility (ICF)	0% (0)	0% (0)	
Another type of facility	21.4% (150)_c_	9.7% (540)_c_	
Home Health Care (HHC)	28.6% (200)_b_	17.5% (975)_b_	
Against medical advice (AMA)	0% (0)	2.5% (140)_d_	
Died	0% (0)	0.4% (20)_a,d_	

* Statistically significant, each subscript letter denotes a subset of disposition of discharges whose column proportions did not differ significantly from one another at *P* < .05.

## Discussion

This study represents the largest, nationally representative analysis to date examining the discharge level occurrence rate, risk factors, and outcomes associated with facial nerve palsy in patients hospitalized for MOE within the United States. Facial nerve palsy was present in approximately 1 in 8 hospitalizations for MOE. Although our findings reflect a discharge‐level estimate rather than a true incidence rate since repeat hospitalizations by the same patient cannot be distinguished in the NIS, our discharge‐level estimate is slightly lower than incidence rates reported in international retrospective chart reviews, which range from 14.9% to over 30%.^14^, ^15^, ^16^ These differences may also reflect regional differences in patient populations, inpatient versus outpatient study settings, disease severity at presentation, or diagnostic thresholds for facial nerve palsy.

We found that stays with facial nerve palsy were more likely to occur in older individuals and in males than those without palsy. Several comorbidities were also more likely to occur in hospitalizations involving palsy, including diabetes with chronic complications, complicated and uncomplicated hypertension, and peripheral vascular disease. These suggests that hospitalizations with palsy may be more frequent in individuals with greater burden of chronic diseases.

Though stays with facial nerve palsy were more likely to have diabetes with chronic complications, they were not more likely to have diabetes without chronic complications. This is an interesting finding, as diabetes has been identified as an important risk for MOE and for facial nerve palsy within MOE, but previous studies have not differentiated between types of diabetes.^23^, ^24^ Our findings suggest that the observed association between diabetes and facial nerve palsy may be driven by diabetes severity or microvascular complications, rather than diabetes itself.

Hospitalizations with palsy also had higher mean counts of both diagnoses and chronic conditions, underscoring an association with an elevated comorbidity burden relative to stays that did not involve facial nerve palsy. Whether this associated burden is a predisposing factor for facial nerve palsy, a consequence of worse health status prior to MOE, or both, cannot be determined from our retrospective cross‐sectional dataset. These findings highlight that comorbidity burden might be a risk factor associated with facial nerve palsy in patients with MOE and warrant further causal analysis.

Facial nerve palsy was independently associated with longer length of stay. In the adjusted log‐transformed model, hospitalizations with facial nerve palsy had an estimated 28% longer length of stay, indicating increased likelihood of greater inpatient resource utilization. This finding is consistent with a study utilizing the Nationwide Readmissions database, which reported that cranial neuropathies in patients with MOE were associated with increased likelihood of readmission and prolonged hospital stays.^25^ Prolonged hospitalization likely reflects greater disease severity and complexity of care and also implies increased healthcare costs.^26^

Facial nerve palsy was also associated with a lower proportion of discharges home. These hospitalizations were more likely to require home health services or transfer to another facility. Although deaths and short‐term hospital transfers were uncommon in both groups, the higher proportions of hospitalizations with facial nerve palsy that required post‐acute care may entail a greater burden of illness and more complex recovery trajectories.

In addition to reflecting greater healthcare needs, non‐home discharge destinations may have larger implications for long‐term outcomes. A retrospective study utilizing another large, US‐based database of common surgical procedures found that non‐home discharges were associated with increased risk of unplanned readmission and mortality, as well as post‐discharge infectious complications, pulmonary infections, deep venous thrombosis, and bleeding requiring transfusions.^27^ Another Japanese study of patients hospitalized for congestive heart failure reported that those discharged to non‐home destinations had higher 1‐year all‐cause mortality rates.^28^ A study of patients with head and neck cancer in Pennsylvania found that patients discharged to a skilled nursing facility or to home health were likely to have four or more comorbidities and were more likely to be readmitted than patients with head and neck cancer.^29^ While those studies did not examined patients admitted with MOE, these findings suggest that the higher proportion of observed non‐home discharge in this population could reflect an elevated risk profile and may portend worse long‐term outcomes. Poorer outcomes from non‐home discharge could also be reflective of patients with more comorbidities requiring more care and being more likely to be discharged to an assisted setting.

We also observed geographic and hospital‐size variations in where hospitalizations with facial nerve palsy. Stays involving facial nerve palsy were more common in the Midwest and West and were less common in the South, suggesting regional variation in outpatient treatment or disease severity at presentation. Stays involving palsy also were more frequent in large hospitals, potentially reflecting availability of neurotologists or greater diagnostic and treatment capabilities within larger centers.

This study has several limitations. Reliance on ICD coding may lead to misclassification of MOE or facial nerve palsy. The NIS does not capture the timing of palsy onset, outpatient course, functional recovery, House‐Brackman grading, or laboratory values that may be relevant to clinical severity. We cannot determine whether facial nerve palsy was present on admission, developed during hospitalization, or occurred following a surgical intervention. Although procedure codes allow identification of inpatient ear surgeries, the NIS does not specify whether the facial nerve palsy diagnosis preceded or followed these procedures. Since both advanced MOE and operative management are potential contributors to facial nerve palsy, we retained these cases in our analysis; however, some may represent iatrogenic palsy.

Because disposition is a multinomial outcome and the NIS lacks patient‐level identifiers, most analyses were necessarily univariate, and all analyses reflect stay‐level rather than patient‐level associations. These findings should therefore be interpreted as discharge‐level estimates, which may include repeat admissions for the same patient.

While our analysis included hospital characteristics and some of these were used as covariates in the secondary LOS analysis, most of our results were not adjusted for hospital‐level factors, which could influence management patterns and outcomes in MOE.

Future studies with chart‐level details or prospective design are needed to clarify the temporal relationship between MOE progression, surgical intervention, and palsy onset; evaluate long‐term functional outcomes; and assess healthcare utilization and quality of life. Such work could also distinguish disease‐related palsy from iatrogenic facial nerve palsy, helping determine whether palsy reflects underlying disease severity or serves as a prognostic marker for outcomes.

Despite these limitations, our results provide a nationally representative, multi‐institutional snapshot of facial nerve palsy among MOE hospitalizations in the United States and address an important gap in the literature.

## Conclusions

Approximately 1 in 8 patients hospitalized for MOE within the United States had facial nerve palsy. These patients were significantly more likely to be older, male, and have a higher overall burden of medical comorbidities. Diabetes with chronic complications, but not diabetes without complications, was more common among patients with palsy, suggesting that advanced disease may play a role in cranial nerve involvement. Facial nerve palsy was also linked to longer hospital stays and a greater likelihood of requiring care after discharge.

These findings provide an important first step towards evaluating facial nerve palsy as a potential marker of more severe illness and increased healthcare needs in MOE. They underscore the need for further research to clarify its prognostic value and inform inpatient management.

## Author Contributions

**Emma R. Thompson**: project design, analysis, writing, revising; **Anthony M. Saad**: analysis, writing, revising; **Dr. Sean Z. Haimowitz**: analysis, writing, revising; **Dr. Nour Abdel‐Azim**: analysis, writing, revising; **Dr. Kevin Wong**: conception, revising; **Dr. Andrey Filimonov**: project design, revising.

## Disclosures

### Competing interests

None.

### Funding source

None.

## Data Availability

The datasets used in this research are available for purchase by eligible institutions and individuals at https://hcup-us.ahrq.gov/tech_assist/centdist.jsp.
